# Development on the Proficiency of Diagnosis and Clinical Care for Rare Diseases in Young Physicians in China

**DOI:** 10.1002/hcs2.70042

**Published:** 2025-12-13

**Authors:** Ge Wu, Yongzhang Miao, Siyuan Fan, Wanru Duan, Junyan Qian, Dawei Wu, Eray Yihui Zhou, Zhongli Xu, Jing Xie, Mengchun Gong, Shuyang Zhang

**Affiliations:** ^1^ GMC Lab, School of Biomedical Engineering Guangdong Medical University Dongguan China; ^2^ Peking Union Medical College Hospital Beijing China; ^3^ Xuanwu Hospital of Capital Medical University Beijing China; ^4^ Cancer Hospital Chinese Academy of Medical Science Beijing China; ^5^ National Cancer Center Beijing China; ^6^ Beijing Tsinghua Changgung Hospital Beijing China; ^7^ School of Medicine Tsinghua University Beijing China

**Keywords:** diagnosis and clinical care capabilities, multidisciplinary collaboration, professional training, rare diseases, young physicians

## Abstract

**Background:**

As medical advancements continue and public health levels rise, increasing attention is being paid to the diagnosis and treatment of rare diseases. Given its large population, China's efforts in enhancing overall clinical abilities for rare disease diagnosis and treatment are crucial. However, young physicians still face significant challenges in their professional capabilities in this field.

**Methods:**

Based on interviews with young physicians and medical education experts from rare disease diagnosis and research institutions, this study explored systematic approaches to cultivating and enhancing the expertise of young Chinese physicians in rare disease diagnosis and treatment, along with the necessary support systems.

**Results:**

By thematic analysis, we identified six effective training models: (1) mentorship teaching, (2) specialized training systems, (3) integration of rare disease knowledge into standard curricula, (4) continuous education and career path planning, (5) application of information technology, and (6) institution‐supported international collaboration. We also discussed critical implementation challenges such as resource intensity and interdisciplinary friction.

**Conclusion:**

Based on these findings, we proposed a series of targeted strategies and recommendations. This study provided valuable experiences for young physicians in clinical and research institutions nationwide, thereby improving the overall level of rare disease diagnosis and treatment.

## Graphical abstract

This figure presents a six‐pillar conceptual framework detailing key strategies—including mentorship, specialized immersion, integration with curricula, continuous career development, and international collaboration—to enhance proficiency in rare disease diagnostics and clinical care among young physicians in China.

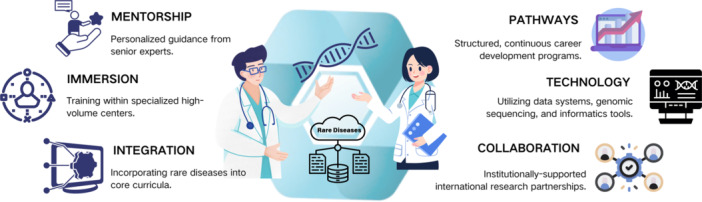

**Abbreviations**
NHS: National Health Service
NRDRS: National Rare DiseaseRegistry System of China

## 1 Introduction

Rare diseases, characterized by low incidence and high clinical heterogeneity, encompass hereditary, nonhereditary and syndrome types, posing higher demands on the medical system. Many developed countries have promoted the development of this field through legislation support, orphan drug research, and systematic diagnosis and treatment studies. Some of these countries have effectively supported rare disease research and physician training through legislation and financial means, promoting the development of young physicians in this field. For instance, the European Rare Disease Plan of the European Union [1], the rare disease services provided by the National Health Service (NHS) in the UK [2, 3], and the research funds, support for clinical trials, and educational and internship opportunities for medical students and young physicians established by Canada through its rare disease policy framework [4]. These practices in various countries fully demonstrate that enhancing the medical skills of young physicians can significantly improve the quality of life and treatment outcomes of patients with rare diseases. However, in China, the capacity building and training of young physicians in this area still need to be further optimized.

China has a large population, and the number of patients with rare diseases is also considerably large. With the increasing awareness of rare diseases in society, the demand for high‐quality medical services from patients is also growing. Although the research, diagnosis, and treatment of rare diseases in China started relatively late compared with developed countries, the number of related research institutions and projects has gradually increased, and the training and participation of young physicians have also been continuously improving, owing to the introduction of multiple supportive policies as well as the increasing social attention [5]. However, the lack of professional knowledge of rare diseases in less developed areas, insufficient diagnostic techniques, and the absence of a systematic training mechanism pose great challenges to the development of rare diseases in China. Therefore, it is of great practical significance to explore and propose suggestions for the cultivation and improvement of the diagnosis and treatment capabilities of young physicians in rare diseases.

This study analyzed the characteristics and current challenges of rare disease diagnosis and treatment in China, and the successful experiences in the training of young physicians. We proposed some effective strategies for cultivating and enhancing the diagnosis and treatment capabilities of young physicians in rare diseases in China, and discussed how to improve the professional capabilities of young physicians through the combination of scientific research and clinical practice.

## 2 Methods

### 2.1 Study Subjects

A qualitative case study approach was employed to gain in‐depth insights into successful training models. A purposive sampling strategy with maximum variation was used to select six representative cases. This strategy was chosen to capture a wide range of experiences across key dimensions that influence rare disease training in China, including: (1) Institution level: cases were selected from national medical centers, university‐affiliated hospitals, and specialized research institutes to represent the top tier of rare disease care and education; (2) Medical specialty: cases covered diverse specialties such as neurology, genetics, surgery, dermatology, and obstetrics, where rare diseases are frequently encountered; (3) Training model: we aimed to include cases that exemplified different pedagogical approaches, such as mentorship, specialized rotations, and integrated curricula. (4) Career stage: interviewees ranged from medical students and residents to attending physicians and research fellows. The selected cases are therefore not statistically representative but are information‐rich exemplars that collectively provide a comprehensive overview of the emerging best practices in rare disease training within China's leading institutions. We analyzed the problems faced by young physicians in clinical skills training and the underlying reasons through their experience during practice, thereby providing useful references and strategies for improving the effectiveness of clinical skills training for young physicians.

This study employed the case study method. The selected cases were all derived from actual medical practices and educational training activities, including multi‐dimensional interview subjects such as senior physicians engaged in rare disease work, professional researchers, and doctoral students. These cases cover the process from the initial contact with rare disease‐related work to their full engagement. Through specific examples, the implementation effect of training strategies and their impact on improving the abilities of young physicians were demonstrated. The institutional attributes of the interview subjects, their professional backgrounds, and the training stages they are in are shown in Table 1.

**Table 1:** Basic information list of interviewed subjects.

Case	Institutions	Clinical speciality	Titles	Clinical and training experience	Experience in primary‐level hospitals	Cases of treating rare diseases (per year)
1	Specialized national clinical medical research center	Neurosurgery	Attending doctor	13 years of clinical experience; Trained via mentorship teaching model	None	~50
2	National medical center	Neurology	Attending doctor	6‐year experience of attending doctor; Trained via mentorship teaching model	None	~800
3	First‐rate university	Clinical medicine major	Doctoral student	11 years of clinical experience and 6 years experience for rare disease; Trained via mentorship teaching model, clinical training, MDT clinics training and workshop	1 year	Not Applicable
4	National medical center	Rheumatology and immunology/internal medicine	Attending doctor	15 years of clinical experience; Training via mentorship teaching model	About 2 years	~60
5	Hospital affiliated to a comprehensive university	Dermatology	Attending doctor	1 year of clinical experience; Trained via mentorship teaching model	None	~20
6	National medical center	Medical oncology	Associate professor	11 years of clinical experience; Trained via mentorship teaching model	About 1 month per year, a total of 12 months to date	~50

### 2.2 Interview Outline and Content Extraction

The core issue focused on: “How to build a systematic and practical training model to comprehensively enhance the professional capabilities of young physicians in rare disease diagnosis and treatment and related fields?” This issue aims to create a sustainable improvement mechanism through multi‐dimensional strategies, including tapping resources, leveraging hospital systems, participating in international exchanges, optimizing the education system, and integrating multi‐disciplinary knowledge with the application of advanced technologies.

For the interview content, we used thematic analysis to code and summarize the responses of the case interviews to extract the core contents of rare disease diagnosis and treatment capability training for young physicians. We conducted line‐by‐line coding of the interview texts, of six cases, extracting key words (such as “mentorship guidance”, “interdisciplinary rotation”, “international research projects”). Through repeated comparison and summarization, similar codes were merged into secondary themes, and six core themes related to “capability training on rare disease” were summarized (Table 2).

**Table 2:** The mapping relationship between open code, secondary themes, and core themes.

Examples of extracted key words (open code)	Secondary themes	Core themes
Mentorship guidance; one‐on‐one guidance by experts; mentorship in scientific research cooperation	Personalized experience delivery	Mentorship teaching model
Professional courses; clinical practice‐based training	Systematic knowledge integration	Specialized training system for rare disease diagnosis and treatment
Integrating rare disease knowledge into medical courses; clinical rotation	Combination of basic and clinical medicine	Integrating rare disease knowledge into basic and clinical training
Residency training program; continuous education for rare diseases	Career development path planning for clinicians	Continuous education and career development path planning
Establishment of structured medical records; application of a registration system for rare diseases	Informatization and data‐driven	Effective utilization of information technology and the case registration system
Participation in international conferences; international research projects	International collaboration	Institutionally supported international collaboration in rare disease research

### 2.3 Data Collection and Analysis

Semi‐structured interviews were conducted using a flexible guide that covered key topics: the physician's journey in rare disease, description of their training experience, perceived strengths and weaknesses of the training model, and suggestions for improvement. This format ensured that all relevant topics were covered while allowing interviewees to introduce unanticipated themes. Each interview lasted between 20 and 30 min. With participants' consent, interviews were audio‐recorded and professionally transcribed verbatim to ensure accuracy. Field notes were also taken during and immediately after each interview to capture contextual observations.

Thematic analysis followed the six‐phase framework. (1) Familiarization: researchers repeatedly read the transcripts to immerse themselves in the data; (2) initial coding: two researchers independently conducted line‐by‐line coding of the first three transcripts to generate initial codes (open coding). An initial codebook was developed; (3) theme development: the researchers then compared their codes, discussed discrepancies, and refined the codebook through an iterative process to achieve consensus. This codebook was used to code the remaining transcripts (Table 2); (4) reviewing themes: the preliminary themes were reviewed against the coded data and the entire data set to ensure they formed a coherent pattern; (5) defining and naming themes: each theme was clearly defined and named to capture its essence; and (6) producing the report: the final analysis was woven into the results and discussion sections.

The independent coding by two researchers and subsequent discussion to reach consensus enhanced the trustworthiness of the analysis and mitigated individual coder bias. Data saturation was considered achieved when subsequent interviews no longer yielded new themes or insights relevant to the research question, which occurred after the fifth interview. The sixth interview confirmed the saturation.

## 3 Results

Through in‐depth analysis of the six cases, we identified and synthesized six effective training models for enhancing the proficiency of young physicians in diagnosing and managing rare diseases. These models are not mutually exclusive but are often interwoven in practice, collectively addressing the multifaceted challenges in rare disease care. The following subsections detail each theme, supported by illustrative data from the case interviews.

### 3.1 Theme 1: Mentorship Teaching Model

The one‐on‐one mentorship model was identified as a fundamental component for cultivating deep expertise in rare diseases. This theme is characterized by personalized guidance from senior experts, facilitating immersive learning through direct collaboration in both clinical and research settings, which is one of the most common models for training young physicians in China.

A representative case involved a young neurosurgeon (Case 1) who, under the supervision of a dedicated mentor, participated in the full continuum of care for a patient with osteogenesis imperfecta. His engagement spanned from foundational research to surgical planning and execution. This guided, hands‐on experience was instrumental in his successful independent completion of a complex surgical intervention, significantly enhancing his capability to manage rare genetic bone disorders [6]. The model's effectiveness is further evidenced in Case 4, where a resident physician received structured training within a formalized “ladder” system. Learning under mentor guidance, the physician developed the competence to lead a multidisciplinary consultation for a critical case of systemic lupus erythematosus complicated by pulmonary hypertension during pregnancy.

Analysis of these cases indicated that the mentorship model effectively transfered specialized knowledge and practical skills. It provides a supportive framework for young physicians to gradually assume greater responsibility, thereby building confidence and fostering the ability to independently manage complex rare disease cases. This finding aligns with international studies emphasizing the critical role of mentorship in fostering clinical expertise and research skills in rare diseases [4].

### 3.2 Theme 2: Specialized Training System for Rare Disease Diagnosis and Treatment

Immersion within a specialized rare disease center emerged as a powerful model for accelerated learning. This theme centers on leveraging high‐volume clinical exposure and integrating research activities into practical training, with patients serving as key educational resources.

An example came from a young neurologist (Case 2) who utilized a dedicated digital platform for encephalitis. Through this system, he managed a high volume of complex cases and participated in the construction and long‐term management of a disease‐specific patient cohort. This immersive experience enabled the rapid accumulation of expert‐level clinical skills and directly led to the publication of multiple research outputs, demonstrating the model's success in bridging clinical practice with academic research. The value of this systematic approach lies in its structured environment. It provides focused exposure to a concentrated number of rare disease cases, which is crucial for overcoming the inherent challenge of low incidence. Furthermore, it formally involves trainees in data collection and cohort studies, thereby cultivating essential skills in both clinical management and research methodology simultaneously.

Analysis indicated that this systematic, immersion‐based training effectively built diagnostic confidence and treatment proficiency by providing repeated, hands‐on experience in a supported setting. This case also highlighted the positive impact of cross‐disciplinary training on improving the comprehensive diagnosis and treatment capabilities of young physicians for rare diseases.

### 3.3 Theme 3: Integrating Rare Disease Knowledge Into Basic and Clinical Training

The integration of rare disease knowledge into foundational training was identified as a critical strategy for building early competence. This theme focused on weaving knowledge of rare disease into the core curriculum of medical training, from basic medical courses to clinical rotations [7].

This approach was observed in the joint training program of Tsinghua University and Peking Union Medical College Hospital (Case 3). The curriculum incorporated the etiology and mechanisms of rare diseases into basic science subjects like biochemistry and genetics through dedicated lectures. Furthermore, clinical rotations provided direct exposure to rare disease cases in multidisciplinary clinic settings, allowing students to gain practical diagnostic experience under expert supervision. The model's validity is reinforced by its alignment with international standards. Major licensing examinations, such as the United States Medical Licensing Examination, include rare disease content to assess understanding of pathophysiology and genetic mechanisms [7, 8].

Analysis indicated that this integrated approach ensured a cohesive educational experience. It builds a robust knowledge framework from the outset of a physician's training, preparing them to recognize and understand rare diseases before encountering them in independent practice.

### 3.4 Theme 4: Continuous Education and Career Development Path Planning

A structured, laddered training model was identified as essential for sustaining long‐term professional growth in rare disease care. This theme encompasses formalized residency programs, postdoctoral training, and continuous learning opportunities, all supported by mentorship and multidisciplinary collaboration [9].

This model is exemplified by a physician (Case 4) trained through the “Clinical Medicine Postdoctoral Program” at Peking Union Medical College Hospital. The program provided a clear career development path, featuring rotations across diverse clinical settings, one‐on‐one mentorship, and seminars for complex case discussions. A critical test of this training occurred when the physician managed a high‐risk pregnant patient with systemic lupus erythematosus and pulmonary hypertension. The ability to promptly organize a multidisciplinary consultation and formulate an effective treatment plan, resulting in a successful 7‐week extension of pregnancy and delivery, directly demonstrated the efficacy of this continuous, structured training approach [10].

Analysis indicated that this career‐long developmental model systematically addressed the challenge of low case exposure. It provides a learning environment where physicians progressively build expertise, from foundational skills to the independent management of highly complex, critical cases, ensuring preparedness for the entire spectrum of rare disease challenges.

### 3.5 Theme 5: Effective Utilization of Information Technology and Case Registration System

The strategic application of information technology and data systems was identified as a key enabler for standardizing rare disease care and enhancing diagnostic precision. This theme highlights the critical role of structured data collection, registry platforms, and bioinformatics tools in supporting clinical decision‐making and research.

This approach was demonstrated in dermatology (Case 5), where a department‐led initiative established standardized, structured medical records for rare skin diseases at both departmental and hospital levels. This created a unified data infrastructure. Furthermore, collaboration with academic institutions provided physicians with training in bioinformatics, enabling them to apply omics technologies in their diagnostic work.

The analysis showed that this model transformed isolated clinical encounters into structured, analyzable data. The implementation of a standardized registration system not only aids in the immediate clinical management of patients but also generates high‐quality data. This creates a valuable foundation for subsequent epidemiological research and contributes to the building of larger, more robust rare disease cohorts. By formalizing the diagnostic process and integrating computational tools, this model enhances both the accuracy of individual patient diagnoses and the overall research capacity of the institution.

### 3.6 Theme 6: Institutionally Supported International Collaboration in Rare Disease Research

Formal institutional support for international engagement was a defining characteristic of high‐level training. This theme underscores the role of structured programs in providing access to global expertise, advanced technologies, and collaborative research projects.

A key example involved an oncologist (Case 6) whose institution facilitated a partnership with a leading European center. This collaboration focused on a specific rare cancer and included standardized data collection, joint analysis of genetic sequencing results, and coauthorship of publications. An accompanying exchange program enabled the physician to spend 3 months at the partner institution, gaining hands‐on experience in advanced diagnostics. This direct immersion not only enhanced the physician's personal expertise but also led to the establishment of a specialized local clinic, directly improving domestic patient care capacity.

Furthermore, many medical universities offer young physicians the opportunity to participate in rare disease research projects through cooperation with research institutions in various countries, and conduct scientific research training under the guidance of mentors, thereby enhancing their research capabilities and clinical diagnosis and treatment levels [11].

Analysis showed that this model transcended simple knowledge acquisition. It embeds young physicians within global research networks, accelerating their understanding of international standards and best practices. The model's primary value lies in its ability to translate international expertise into tangible local improvements in clinical practice and research infrastructure, elevating the overall standard of care.

### 3.7 Critical Challenges and Obstacles in Implementation

While the cases above highlight successful strategies, our interviews also revealed significant challenges and barriers that can hinder the effective implementation of these training models, even in well‐resourced institutions.

A predominant theme was the substantial investment of time and resources required. The “one‐on‐one“ mentorship model (Case 1) and multidisciplinary consultations (Case 4) were noted as particularly demanding on senior experts' time, creating scalability issues. Participants reported that initiating and maintaining a structured rare disease registry (Case 5) faced initial resistance due to the additional documentation burden on clinicians, requiring dedicated data management support to overcome. Furthermore, interdisciplinary collaboration, while highly beneficial, often encounters practical friction. Differences in professional terminology, diagnostic priorities, and workflow patterns between departments could lead to communication gaps and slow down decision‐making processes, underscoring the need for dedicated coordination and established communication protocols. In addition, sustaining trainee engagement posed a challenge. The long diagnostic odyssey and emotional toll associated with rare diseases, coupled with the pressure to meet general clinical productivity targets, sometimes led to burnout and attrition among young physicians initially interested in the field. This suggested that effective training models should be coupled with strong institutional support and career incentives to maintain long‐term commitment.

## 4 Discussion

### 4.1 Training Models for Young Physicians' Rare Disease Diagnosis and Treatment Capabilities and Their Advantages

This study underwent case studies of multiple rare disease field practitioners. We summarized six current training strategies for young physicians' rare disease diagnosis and treatment capabilities in China, which were the mentorship teaching model, specialized training system for rare disease diagnosis and treatment, rare disease knowledge integrated into basic and clinical training, continuous education and career development path planning, application of information technology, and international collaboration. Each model has its unique advantages. In the “one‐on‐one” mentorship teaching model, the mentor can provide personalized guidance and feedback for young physicians. The specialized training system has a systematic course design, combining theory with practice, as well as multidisciplinary collaboration. The basic and clinical training emphasizes the combination of basic medicine education and clinical practice, which can enable medical students to comprehensively learn the knowledge of rare diseases, providing a deeper understanding of disease mechanisms for young physicians. The continuous education and career development path planning model is physician‐led, and a clear career development path and continuous educational support can motivate the long‐term development of young physicians, providing different levels of training and learning opportunities for physicians of different seniorities. The application of information technology can improve diagnosis and treatment efficiency and quality, and disease registration systems also facilitate data collection and analysis. Based on these models, taking advantage of international exchange opportunities provided by institutions can broaden the horizons of young physicians and facilitate discussions on the latest research in rare diseases.

### 4.2 Problems in the Training of Young Physicians' Rare Disease Diagnosis and Treatment Capabilities

The main problems that young physicians encounter in the training of rare disease diagnosis and treatment capabilities include uneven distribution of medical education resources, limited opportunities to gain practical experience, lack of systematic training plans, and insufficient research support [12]. These problems often lead to a lack of confidence and direction when young physicians face rare disease cases, making it difficult for them to make accurate diagnoses and effective treatments, and also affecting their career and academic development.

Although the current medical education system in China provides a certain amount of basic medical knowledge and general clinical skills training, it appears insufficient in specialized education and clinical practice in the specific field of rare diseases [13]. In addition, the current education and medical system pays insufficient attention to the individual differences of young physicians and lacks specialized training for the characteristics of rare disease diagnosis and treatment [14]. To address these multifaceted challenges, a set of targeted strategies is proposed. The uneven distribution of resources necessitates institutional‐tailored models. The lack of systematic training calls for reforms in medical education and structured continuing education pathways. The complexity of rare diseases demands the construction of multidisciplinary platforms and support for innovative research, which can also help alleviate the pressure between clinical work and scientific research output.

### 4.3 Suggestions for Improving the Rare Disease Diagnosis and Treatment Capabilities of Young Physicians in China

#### 4.3.1 Improvement and Reform of the Medical Education System

To enhance the rare disease diagnosis and treatment capabilities of young physicians, it is necessary to improve the medical education system. This includes adding rare disease‐related teaching content in medical school curricula to ensure that medical students have a certain understanding of rare diseases, as well as the basic knowledge and initial diagnosis and treatment skills of rare diseases. Curriculum reform and the improvement of the teaching system are of great significance to the specialized training system model and the continuous education and career development path planning model. Meanwhile, clinical practice training should be strengthened, encouraging students to participate in the actual diagnosis and treatment of rare disease patients, and the course content should be constantly updated based on the latest research and guidelines of rare diseases. Besides, due to the relatively small number of rare disease cases, teaching with simulated cases could also be added to the courses to enhance the clinical thinking and problem‐solving abilities of young physicians.

#### 4.3.2 Construction of Multidisciplinary Collaboration Platforms

**Figure 1:**
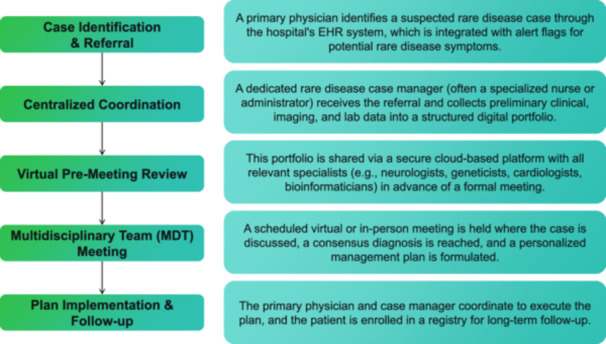
Workflow for multidisciplinary team (MDT) collaboration in rare disease management. The process includes: (1) case identification and referral, (2) centralized coordination with digital portfolio creation, (3) virtual pre‐meeting review, (4) MDT meeting for consensus, and (5) plan implementation and follow‐up with registry enrollment.

The complexity of rare diseases requires physicians to have a multidisciplinary knowledge structure and collaboration ability. It is crucial to establish a multi‐disciplinary collaboration platform involving medical institutions, universities, research institutes, enterprises, and government departments [15]. The establishment of such a platform requires efforts in multiple aspects, including forming multi‐disciplinary teams, establishing collaboration mechanisms, and improving support systems. Rare disease experts, specialized nurses, genetic counselors, psychiatrists, data experts, medical social workers, and administrative staff should be involved in the multi‐disciplinary teams for their corresponding duties, including diagnosis and treatment decisions, daily care and education of patients, collection and analysis of data, providing psychological support for patients, and logistics and administrative work of the team. Moreover, the composition of multidisciplinary teams should be expanded beyond medical professionals to include representatives from patient advocacy groups. These groups provide invaluable insights into the lived experience of the disease, contribute to designing patient‐centric care pathways, and can play a crucial role in educating healthcare professionals about the daily challenges and unmet needs of patients and their families. Regarding the construction of the collaboration platform, cloud computing, big data, and other technologies can be applied, facilitating the sharing of case data, research results, meeting minutes, and other information, and supporting functions such as remote consultation, case discussion, and academic conferences. Based on our case experiences, a practical workflow for multidisciplinary collaboration can be schematized (Figure 1). In terms of communication mechanisms, multidisciplinary meetings can be arranged regularly for case discussion sessions and research progress sharing to promote knowledge exchange and idea collision among team members. For difficult cases or research projects, temporary or long‐term working groups can be established to focus on those problems. Moreover, an evaluation mechanism can be established to measure the effectiveness of collaboration, including treatment outcomes, patient satisfaction, team collaboration efficiency, and other indicators, which can improve the collaboration and make corresponding adjustments.

The multidisciplinary collaboration platform is one of the effective forms of applying information technology to train young physicians. And it can also be beneficial to other training models, providing a good platform and opportunity for young physicians to get involved in rare disease‐related work. Our proposed approach was consistent with international evidence that MDT collaborations were paramount for improving diagnostic yields and patient outcomes in rare diseases [15].

#### 4.3.3 Support Mechanisms for Innovation and Research

Encouraging young physicians to participate in innovative research on rare diseases plays a crucial role in enhancing their professional capabilities. Medical institutions and academic organizations should establish special funds to support young physicians in conducting research projects related to rare diseases and provide them with the necessary research resources. These policy preferences can not only promote their abilities in the integration of basic and clinical knowledge, but also encourage young physicians to participate in international collaborations in the form of innovative research. Additionally, with the development of new technologies such as large language models (LLMs), opportunities and challenges have emerged in the field of rare diseases. Currently, although there are still significant gaps for artificial intelligence like GPT and DeepSeek to be applied in the healthcare field [16], these LLMs have demonstrated the potential of clinical decision support in rare diseases [17]. Looking forward, emerging technologies such as LLMs hold potential for creating innovative training tools (e.g., diagnostic simulators, knowledge retrieval systems). Future research should explore the feasibility and effectiveness of such AI‐assisted learning modules in the specific context of rare disease education in China, which provides opportunities for young physicians to further study rare diseases and integration with advanced AI technology.

Although current LLMs still have limitations in their application within the medical field (including issues like hallucinations and a lack of clinical validation), they have demonstrated potential value in assisting education and training for rare diseases. We propose the following AI‐assisted training solutions: (1) build a rare disease knowledge base and Q&A system: Integrate domestic and international guidelines, case reports, imaging, and pathology data to construct localized knowledge graphs, enabling junior doctors to query and learn; (2) virtual case simulation system: leverage AI to generate typical or atypical virtual cases of rare diseases for diagnostic reasoning training, supplemented by feedback mechanisms providing explanations and suggestions; (3) diagnostic assistance tool: embed AI‐powered prompt features into electronic health record systems to help physicians identify potential rare disease clues during consultations, reducing missed diagnoses and misdiagnoses. These tools should serve as adjuncts rather than replacements for clinical decision‐making, requiring supervision by attending physicians and validation against real‐world cases. Emerging research indicates that AI holds promise in areas such as rare disease image recognition and phenotype matching [16, 17], though broader preclinical and clinical studies are needed to support its widespread implementation.

#### 4.3.4 Continuing Education and Career Development Path Planning

Continuing education is an important way to enhance the professional skills of young physicians. Since it lacks rare disease‐related training in medical education in China, this model may be suitable for most young physicians who are already working on the clinical front line. A continuing education plan contains various forms, such as online courses, workshops, and academic conferences, to meet the needs of young physicians at different career stages. Moreover, it is necessary to build a multi‐level training system. At the policy level, rare disease diagnosis and treatment ability can be considered as an evaluation index of specific physician promotion. Besides, the programs should formally incorporate the perspective of patient advocacy groups. Inviting patients and family members to share their diagnostic odysseys or including modules developed in partnership with patient organizations can profoundly enhance young physicians' empathy and understanding of the psychosocial dimensions of rare diseases, complementing their clinical training.

Incorporating rare disease diagnosis and treatment capabilities into physician promotion evaluation systems indeed faces conflicts with the traditional research paper‐dominated assessment framework. To address this, we propose the following measures to achieve a balance between clinical practice and scientific research: (1) Establish specialized clinical competency channels: create dedicated pathways for evaluating “Rare Disease Diagnosis and Treatment Capabilities” within professional title reviews. Comprehensive assessments could utilize multidimensional materials such as case reports, treatment plan designs, and patient follow‐up outcomes; (2) Transform clinical practice into research output: Encourage clinicians to convert clinical cases into research data through initiatives like registries, cohort studies, etc. This dual approach enhances diagnostic expertise while generating scholarly contributions; (3) Policy support and resource allocation: Health administration departments should collaborate with hospitals to establish specialized funds for rare disease clinical research, providing seed funding and protected time allocations for physicians engaged in this study. International experience demonstrates that integrated evaluation systems combining clinical competence with research excellence better serve long‐term development in rare diseases [4, 8]. China could draw inspiration from successful models like Canada's FORGE program [4], leveraging policy guidance and institutional innovation to foster synergy between clinical care and scientific advancement.

**Figure 2:**
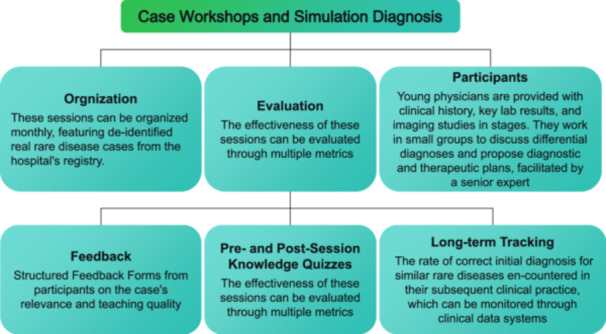
Organization and evaluation of the “Case Workshop and Simulation Diagnosis” session. Monthly sessions use real deidentified cases. Young physicians work in small groups with expert facilitation. Evaluation combines knowledge quizzes, participant feedback, and long‐term tracking of diagnostic accuracy.

At the practical level, continuous training can be enhanced through structured “case workshop and simulation diagnosis and treatment” sessions (Figure 2), whereas senior physicians can participate in multidisciplinary consultation and international academic conferences to strengthen their ability to deal with complex cases. In terms of incentive mechanisms, special funds can be set up to support young physicians to go to a national medical center for further study. Besides, the field of rare diseases can be favored in scientific research project approval and academic evaluation, so as to enhance the career attractiveness of young physicians.

#### 4.3.5 Develop Appropriate Training Models for Institutions

The implementation of each training model requires institutional guidance, continuous evaluation, and adequate resource allocation. Due to factors such as uneven distribution of medical education resources and limited opportunities to gain practical experience, different levels of medical institutions (e.g., primary healthcare institutions and national medical centers) should develop appropriate training models and strategies.

For primary healthcare institutions, the key of the training goal should be enhancing the ability to identify rare diseases. The design of the training courses for these institutions could be simplified, focusing on improving young physicians' awareness and identification of rare diseases, as well as their ability in rare disease case management. Online educational resources and remote consultation systems can be utilized to provide basic training for young physicians. For national medical centers, the training of young physicians should focus on in‐depth research and management of complex cases, including participation in clinical trials, genetic diagnosis, and multidisciplinary team collaboration. Advanced training courses and national research projects can be set up to enhance the capabilities of young physicians in the field of rare diseases. Moreover, by establishing a collaborative network between national medical centers and primary healthcare institutions such as community medical institutions and primary hospitals, young physicians can learn from expert experience and advanced diagnostic techniques. Additionally, customized training modules should be designed based on the professional background of physicians to ensure that the training content is closely related to clinical practice.

In addition to the training strategies, it is also crucial to establish a continuous improvement mechanism, including regular assessment of training effectiveness, collection of feedback, and adjustment of training plans. Specifically, for training courses or academic conferences, rare disease course credits and regular assessment mechanisms can be set up to evaluate the training results of young physicians. In terms of clinical practice, the training effect can be comprehensively evaluated through qualitative and quantitative indicator data such as feedback from clinical practice, patient satisfaction surveys, self‐assessments by physicians, and peer reviews. Institutions at different levels can adjust their training strategies based on the evaluation results to ensure that the training model can meet the demands of diagnosis and treatment of rare diseases.

Although the cases in this study primarily originate from top‐tier domestic medical institutions, we acknowledge the current disparity in healthcare resource distribution across regions. Primary medical facilities face greater challenges in building diagnostic and treatment capacities for rare diseases. Consequently, our strategic recommendations prioritize implementing a tiered training framework. For young physicians in primary care settings, training should focus on enhancing early detection skills and referral protocols for rare diseases rather than independent management of complex cases. This can be achieved through telemedicine education, digital case repositories, and collaborative networks linking regional medical hubs—mechanisms designed to channel specialized expertise downward. For instance, the National Rare Disease Registry System (NRDRS) partners with numerous community hospitals, delivering standardized training modules and remote consultation services via cloud platforms. Additionally, drawing inspiration from global best practices (such as clinical network systems that disseminate specialty knowledge), locally adapted models combining medical consortia with disease‐specific alliances could foster knowledge exchange and standardize clinical competencies within integrated health systems.

### 4.4 Limitations

This study has several limitations that should be acknowledged. First, the small sample size of six cases, while providing in‐depth qualitative insights, limits the statistical generalizability of the findings. Second, the selection of interviewees predominantly from leading national medical centers may introduce selection bias, as these institutions possess advanced resources, multidisciplinary platforms, and international collaborations that are not widely available in primary or regional hospitals. Consequently, the proposed training models (such as specialized mentorship programs and international research exchanges) may be less directly applicable in resource‐limited settings. Although we have suggested tailored strategies for different levels of institutions in the discussion, the feasibility and effectiveness of these approaches in less‐resourced environments require further empirical validation. Further research should include a broader range of healthcare contexts, incorporate quantitative evaluations, and explore contextual adaptations of these models to enhance their generalizability across China's heterogeneous healthcare landscape. Third, this study is primarily qualitative in nature, focusing on elucidating the structures and perceived benefits of various training models through rich, narrative data. Therefore, it does not include quantitative metrics (such as improvements in diagnostic accuracy rates, reduction in time to diagnosis, or patient satisfaction scores) to objectively measure the effectiveness of these models. While the cases provide strong anecdotal evidence for the value of these approaches, the absence of such empirical data limits our ability to make definitive claims about their efficacy. Future research should employ a mixed‐methods approach, integrating pre‐ and post‐training assessments, longitudinal tracking of early diagnostic capabilities, and patient outcome measures to quantitatively validate and refine these training strategies.

## 5 Conclusion

Because of the complexity and the relatively small number of cases, the diagnosis and treatment of rare diseases is still a great challenge, especially in developing countries. The uneven distribution of medical and educational resources and technology has limited the systematization and standardization of training in the field of rare diseases. Compared with the training experiences of developed countries, there is still a certain gap in the training of young physicians in rare diseases in China. We summarized six current training strategies in China by interviewing professionals in multiple rare disease fields, and provided certain suggestions for the development of rare disease training, including. Improvement of medical education, integration of multidisciplinary resources, application of information technology, strengthen international academic exchange, and customized courses for different institutions. In addition, enhancing research funds for young physicians is also an effective way to improve their ability in rare diseases. Through these measures, the diagnosis and treatment capabilities of young physicians can be effectively enhanced, and the overall quality of rare disease medical services can be improved.

## Author Contributions

**Ge Wu:** writing – review and editing, writing – original draft, methodology, formal analysis, and visualization. **Yongzhang Miao:** writing – original draft, writing – review and editing, methodology, and formal analysis. **Siyuan Fan:** investigation. **Wanru Duan:** investigation. **Junyan Qian:** investigation. **Dawei Wu:** investigation. **Eray Yihui Zhou:** investigation. **Zhongli Xu:** investigation. **Jing Xie:** investigation. **Mengchun Gong:** conceptualization, funding acquisition, supervision, and project administration. **Shuyang Zhang:** conceptualization, supervision, and project administration.

## Ethics Statement

This study does not involve any biological specimens, related test data, or animal experiments. No procedures in this study posed potential ethical risks to humans, animals, or biological materials.

## Consent

The authors have nothing to report.

## Conflicts of Interest

The authors declare no conflicts of interest.

## Data Availability

The data that support the findings of this study are available from the corresponding author upon reasonable request.
